# The role of chatbots and virtual assistants in enhancing tobacco cessation counselling

**DOI:** 10.3389/fdgth.2025.1503227

**Published:** 2025-04-16

**Authors:** Deepika V, Praveen S. Jodalli, Avinash B R

**Affiliations:** Department of Public Health Dentistry, Manipal College of Dental Sciences Mangalore, Manipal Academy of Higher Education, Manipal, Karnataka, India

**Keywords:** tobacco cessation, artificial intelligence, chatbot, smoking cessation activities, virtual assistant (VA), digital health innovation, tobacco control, health promotion

## Abstract

Tobacco remains one of the leading global public health threats, causing over 8 million deaths worldwide each year. Existing tobacco cessation strategies must be complemented with innovative approaches to enhance their effectiveness. This paper is intended to review the state and success of chatbots and virtual assistants in the delivery of tobacco cessation counselling services. Comprehensive literature search was performed on various databases including PubMed, SCOPUS, EMBASE, COCHRANE Library, and ScienceDirect using relevant keywords concerning tobacco cessation as well as artificial intelligence. The review is limited to studies published between March 2015 and March 2024 examining only the role of chatbots and virtual assistants in smoking cessation. The analysis of 31 studies found that chatbots and virtual assistants deliver continuous support, personalized interaction, and increased accessibility, which collectively boost user engagement. Their anonymity from other means of online counselling has significantly increased the willingness of people who want to stop smoking. The development of information technology has led to the advent of AI-based chatbots and virtual assistants as a promising novel tool for the tobacco cessation process, providing personalized, adaptive, and scalable support to web-based users. Personalized approaches improve the chances of success in quitting smoking and represent a major advancement of cessation strategies.

## 1 Introduction

Tobacco consumption continues to be a leading cause of global public health problems with over 8 million deaths in the year (1). Of these, an estimated 7 million deaths are attributable to direct use of tobacco, and about 1.2 million deaths occur as a result of second-hand smoke exposure (2). Tobacco not only deteriorates health but also leads to increased health care costs and loss of productivity, along with socio- economic consequences. In view of such challenges, the need for tobacco cessation strategies of proven effectiveness is paramount. Quitting smoking greatly decreases the risk of cancer and serious health problems, including heart disease and stroke. Importantly, quitting before age 40 can prevent almost 90% of smoking-related death (3). While these benefits are well documented, the development of more effective cessation tools and programs is an urgent public health imperative.

The integration of technologies such as chatbots and virtual assistants into tobacco cessation programs offers a novel approach to enhancing accessibility and effectiveness of support services (4). These digital tools ensure continuous availability, which is crucial for providing immediate support during cravings or moments of vulnerability, thereby reducing the risk of relapse (5). Chatbots, with their ability to personalize recommendations based on a user's smoking history and past quit attempts, can increase engagement and improve adherence to cessation programs. The anonymity provided by these platforms encourages users to seek help more openly, ask questions without hesitation, and overcome the stigma or embarrassment often associated with seeking smoking cessation support.

Recent studies have shown that AI-based applications are key components to help the smoking cessation (6). Research shows that chatbot-assisted interventions are more highly reported when it comes to satisfaction rates and cessation compared to traditional methods (7). These digital resources offer not only assistance to manage cravings but also knowledge and practical strategies to handle withdrawal symptoms. A 2024 study conducted by (8) found that individuals who employed a virtual assistant as their approach to quitting reached success rates that were 30% higher than more traditional strategies (8).

The objective of this study is to investigate the use and potential of chatbots and virtual assistants for tobacco cessation counselling. It aims to assess how far these technologies contribute to the accessibility, personalization, and efficiency of support to quit smoking. Moreover, this research will explore current limitations and barriers to implementation while making recommendations for health systems administrators, policymakers, and members of the technology industry to optimize these digital innovations to better public health outcomes.

## 2 Methodology

**Figure 1:**
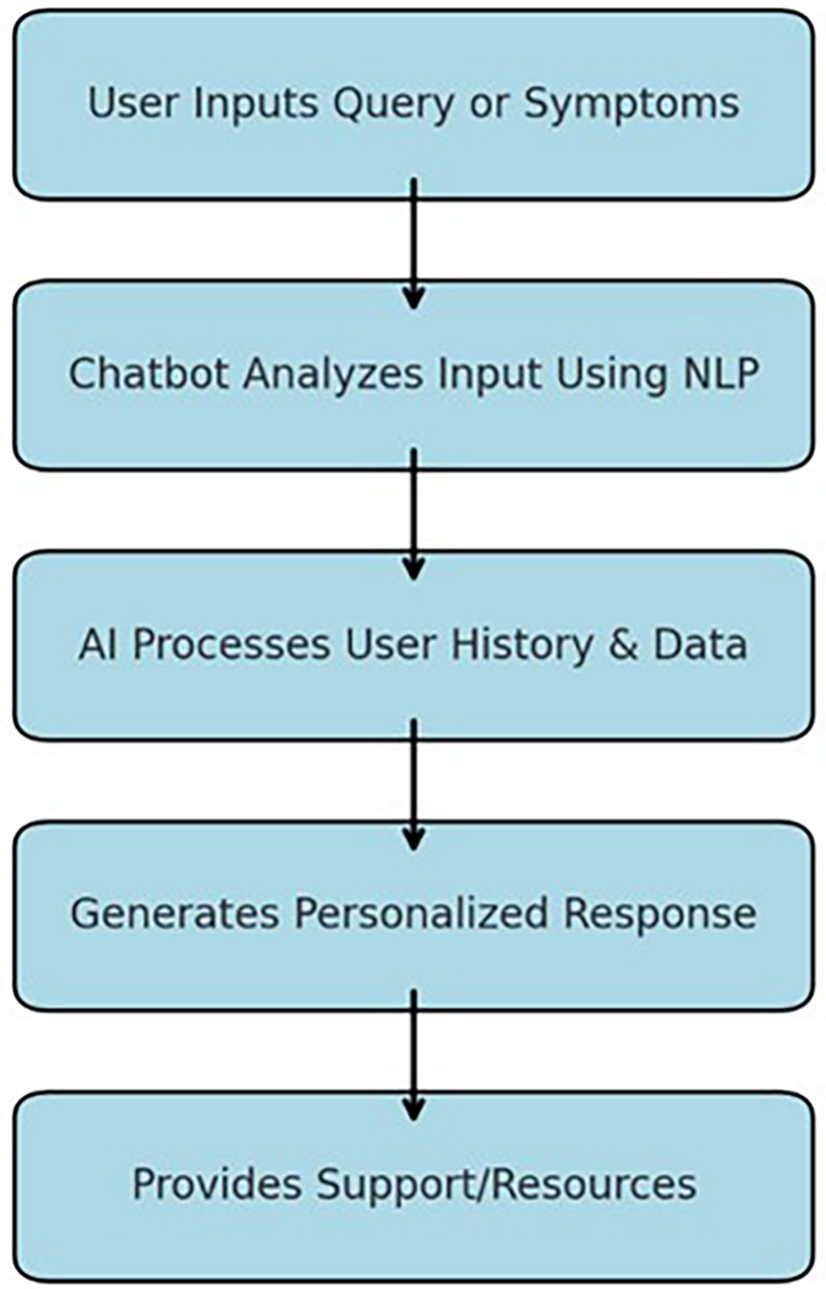
Chatbot based tobacco cessation flowchart.

A systematic electronic literature search was conducted on “The Role of Chatbots and Virtual Assistants in Enhancing Tobacco Cessation Counseling: A Review”, from March to April 2024 in the PubMed, SCOPUS, EMBASE, COCHRANE Library, and ScienceDirect databases. The search included MesH terms and keywords like “Tobacco Cessation”, “Chatbots”, “Virtual Assistants”, “Artificial Intelligence in Healthcare” and “Smoking Cessation”. In addition to electronic searches, cross-referencing and relevant textbooks were reviewed to search for additional articles. A literature search was conducted for articles published in the English language that corresponded to the study objectives during the period between March 2015 and March 2024. Studies were assessed based on predefined inclusion and exclusion criteria, and the quality of the included studies was evaluated. Of the 775 articles that were searched, 67 articles were shortlisted based on their titles and abstracts. After a full-text review and application of inclusion/exclusion criteria, a total of 31 studies were finally included in the review (Figure 1).

## 3 Current tobacco cessation strategies

Evidence based strategies of modern tobacco control programs are combination of interventions that emerged from increasing understanding of medical science, and collective efforts at the public health level. Traditional cessation methods like Nicotine Replacement Therapy (NRT) include patches, gum, and lozenges that help people refrain from smoking. Also, other medications like varenicline and bupropion are used to help combat withdrawal symptoms (9). One effective method is cognitive-behavioural therapy (CBT), which is provided either in individual counseling or group settings, teaching smokers how to cope with triggers and stress (10). In addition, mobile apps and online programs serve as interactive tools that can offer immediate assistance and reinforcement for smoking cessation efforts. A systematic review (SR) conducted by Rigotti et al. (11) stated that the multimodal approach, especially the triplet of counseling and pharmacological treatment, has noticeably better effects on smoking cessation than any single approach.

## 4 Role of technology in health care

The implementation of artificial intelligence (AI) in the healthcare sector has fostered advancements in both the healthcare delivery and system organization. Artificial intelligence is vital for early diagnosis and targeted treatment design by competent analysis of large datasets (12). AI algorithms improve the interpretive accuracy of medical imaging studies (i.e., x-ray, MRI, and other scans) in radiology. They can identify minute pathologies that may fly under the radar of the human eye, thereby increasing the accuracy of diagnosis (13). Machine learning algorithms in the field of oncology aid in predicting how a given patient will respond to different therapeutic regimens of varying types, which is crucial for determining and optimizing patient-tailored treatment strategies (14). Moreover, in the field of mental health, artificial intelligence–powered technologies analyses speech patterns and mood indicators in real time to study possible signs of depression or anxiety disorders and enable early intervention for patients, leading to better results (15).

## 5 Application of chatbots and virtual assistants in tobacco cessation

### 5.1 Mechanisms of action

**Figure 2:**
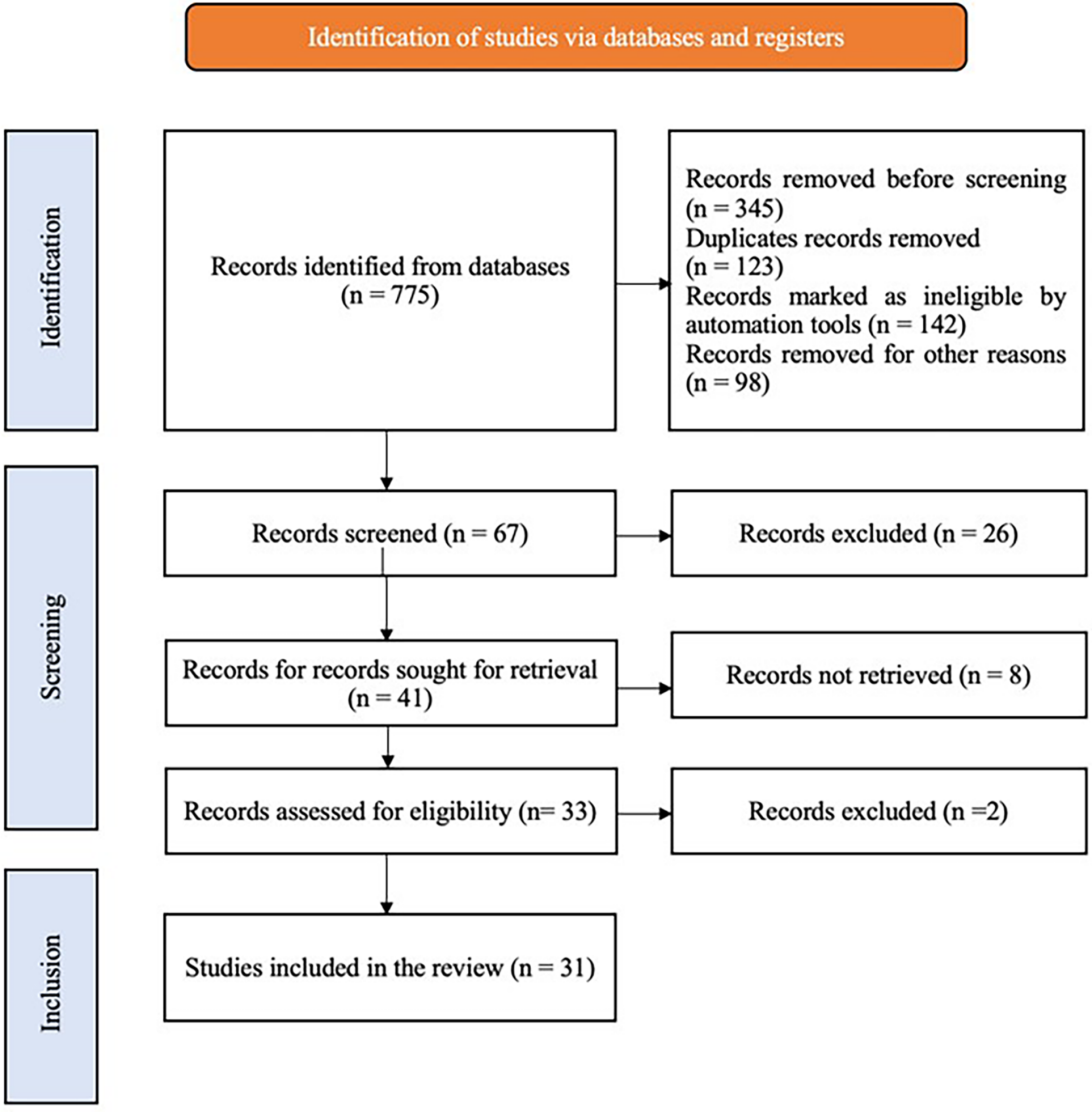
Flowchart showing the step-by-step identification of the studies via databases.

Chatbots and other virtual assistants are innovative approaches to the realm of tobacco cessation as they use AI-based communication to offer immediate intervention and tailored experiences. These digital assistants use natural language processing (NLP) and are therefore easy to use and capable of answering the users' questions (16). They continually gather the client's information, including usage frequencies, smoking history, and past attempts at quitting. This information enables them to prescribe advice and motivational support appropriate to the user's stage of cessation processes (17). For example, a chatbot can suggest that a patient should take medicine or perform an activity that reduces stress when it gets an indication that the user is in a high-risk category based on the user's mood or stress level shared with the chatbot. Such a one-on-one communication approach is useful to address the individuals' hurdles enhancing the probability of a quit attempt (18). The chatbot-based intervention mechanism is illustrated in the flowchart below, highlighting the step-by-step process from user input to personalized support provision (Figure 2).

### 5.2 Accessibility and 24/7 support

Accessibility is one of the key benefits of utilizing chatbots or virtual assistants in smoking cessation. In contrast to human counsellors, these tools are always on, offering constant support when needed specifically for crises, such as cravings or episodes of weakness, which can strike at any time and are unexpected since they occur outside of clinic hours (19). This constant support system can be very important when it comes to relapse because those who are using such an application get assistance and a way to combat cravings anytime they want. That sort of comprehensive, round-the-clock care is very effective in making sure that anyone who wants to quit smoking is never left on their own and has the resources they need close at hand; that way, they do not have to give in to temptation (20).

### 5.3 Privacy and anonymity

Privacy and anonymity are some of the values which would make more people who want to quit smoking to use chatbots and other virtual assistants. This is because the process of quitting may be embarrassing to many people due to the social perception associated with smoking or they may fear the attitudes that a doctor or fellow students may display towards them. Social media provides the user with an environment that is free of judgment, thus clients can freely ask their concerns and get advice without disclosing their identity. This level of privacy increases the level of trust among the users as they can actively participate in cessation programs and be comfortable to share with other users' challenges and relapses which are crucial for support and direction (21).

### 5.4 Recent studies

Recent research and various examples and cases also confirm potentially high efficiency of chatbots and virtual assistants in smoking cessation support. Studying the matter in 2024 (8), reported a 30% improvement in quit rates among the participants with at least one virtual assistant incorporated into the cessation regimen. It shows that these AI tools can have a rather large positive effect on the level of public health by helping smokers to quit successfully. In the findings, the shared interactions and immediate support of a cessation program were highlighted as crucial components in enhancing the general effectiveness of tobacco cessation programs. Consequently, the research points to the fact that incorporating the use of chatbots and virtual assistants into extant conventional cessation models promotes the feasibility of their application, which can be valuable in expanding the use of IT in public health interventions aimed at diminishing smoking prevalence.

### 5.5 Applications and software

Some of the most creative apps and software solutions that include AI technologies have been created to assist a person in the process of smoking cessation. One of the most widely known apps, aimed at quitting the use of tobacco products, is called Smoke Free; this app applies artificial intelligence algorithms for delivering missions and feedback for users, as well as taking into consideration the user's progress and preferences. Quit Genius uses CBT techniques with AI to develop the user's quitting plan and have access to interactive content and real-time support (22, 23). Another important app with significant features is CraveToQuit that incorporates AI in the training of mindfulness to help in managing cravings. Such tools are usually designed to contain elements like the smoking stimuli monitor, motivational push notifications, and a user's connection to a community or a healthcare provider.

## 6 Advantages of chatbots and virtual assistant

A study has revealed that the use of AI tools in helping smokers quit has a higher user satisfaction and a better rate of cessation than traditional approaches like face-to-face counseling or telephone helpline (24). The fact that each interaction with chatbots is unique and users can receive immediate feedback results in higher usage rates of cessation programmes, therefore the programmes' success rate. To each, economically, chatbots and virtual assistants are inexpensive and easy to scale. They can easily manage multiple interactions at once, which means that despite the large number of targeted posts, there is no need to significantly increase the number of employees, thus enabling the organization to cover a large audience and remain accessible, all for a fraction of the cost of traditional methods. The technology utilized in digital health applications can be incorporated into the existing models of care delivery thus assisting the caregivers by offering timely information regarding the patients' outcome (25).

While AI-driven chatbots offer several advantages, it is essential to compare their effectiveness with traditional smoking cessation methods, such as face-to-face counseling and telephone helplines. Face-to-face interactions provide personalized emotional support and deeper engagement, while telephone helplines allow direct communication with trained counselors. However, chatbots and virtual assistants offer 24/7 accessibility, anonymity, and cost- effectiveness, which can encourage more individuals to seek assistance.

Table 1 provides a comparative analysis of chatbot-based smoking cessation interventions vs. traditional methods, highlighting differences in accessibility, personalization, cost, and effectiveness.

**Table 1:** Comparative analysis of chatbot-based smoking cessation interventions versus traditional methods.

Feature	AI Chatbots & virtual assistants	Face-to-face counseling	Telephone helplines
Availability	24/7 support (26)	Limited to office hours (27)	Limited availability (28)
Personalization	AI-based adaptive responses (29)	High (human interaction) (30)	Moderate (31)
Cost	Low-cost and scalable (32)	High (requires human resources) (33)	Moderate (34)
Anonymity	High	Low	Moderate
Effectiveness in long-term engagement	Moderate to high (35)	High (36)	Moderate (37)

## 7 Challenges and limitations

The use of chatbots and virtual assistants in tobacco cessation presents several challenges and limitations that must be addressed. Organizational constraints, such as technical issues, accuracy, and reliability, can affect AI system performance. Natural Language Processing (NLP) limitations may lead to misinterpretation of user inputs, causing frustration or even incorrect advice. Additionally, implementation challenges exist for both healthcare providers and patients—providers may be skeptical about shifting from human-led interventions to automated systems (38–43), while patients may be reluctant to disclose personal health concerns to a machine, slowing down the adoption of AI-based solutions in healthcare. Outside of technical and adoption challenges, the use of AI powered chatbots and Virtual Assistants in healthcare raises significant ethical questions, especially around data privacy, algorithmic bias and accountability. A major concern is the matter of security of personal and health data, where AI systems collect and analyses sensitive information about users, which creates the possibility of unauthorized access and theft of data. Data breaches can have devastating effects, both financially and reputationally, making robust data protection mechanisms critical to ensuring that breaches do not occur, and public trust is not eroded. Algorithmic bias is another significant problem that could introduce disparities in treatment recommendations. An artificial intelligence model trained on a non-representative dataset may be biased, thus leading to less accuracy for some population. AI models should be trained on data that is diverse and representative of the population that the model will serve, to prevent the reinforcement of existing disparities in healthcare.

Accountability is another major neutralization that's legally and ethically complicated — when AI-generated recommendations lead to wrong or harmful outcomes, it can be difficult to ascertain responsibility. Clear regulatory frameworks, transparent AI models, and stringent data security protocols are necessary solutions for these concerns so that responsible AI can be integrated within tobacco cessation counselling.

## 8 Future scope

Discussing the prospects of chatbots and VA in tobacco cessation, it is possible to state that further developments and expansion of their use are expected in the near future. It is also anticipated that the AI tools' effectiveness and credibility will increase over time due to technological advancements in areas such as machine learning and natural language processing which will make AI tools more contextual and hence provide more detailed advice. Policy and implementation strategies will be critical in this process since the policymakers and the healthcare systems will set the framework for safe and ethical practice of AI while at the same time providing incentives for the integration of the AI in the healthcare facilities. The role of the research also continues to be relevant, and one should continue to look for the gaps in the knowledge as for example the long-term impact of AI interventions, ways to attract the users, or the comparison with traditional smoking cessation techniques (39).

## 9 Conclusion

Chatbots and virtual assistants hold significant potential in enhancing tobacco cessation interventions by offering patient-centered, accessible, and cost-effective support. Their integration into public health programs can improve cessation success rates and reach a wider population. However, successful implementation requires addressing technological challenges, ethical concerns related to data privacy and algorithmic bias, and adoption barriers among healthcare providers and patients. Future research should focus on optimizing AI models, ensuring regulatory compliance, and integrating chatbot-based cessation strategies with traditional approaches to enhance overall effectiveness.
